# Innate Immune Response in Brain, NF-Kappa B Signaling and Cystatins

**DOI:** 10.3389/fnmol.2015.00073

**Published:** 2015-12-09

**Authors:** Nataša Kopitar-Jerala

**Affiliations:** Department of Biochemistry, Molecular and Structural Biology, Jožef Stefan InstituteLjubljana, Slovenia

**Keywords:** astrocytes, cystatins, microglia, TLR, NF-kappa B, NLR inflammasome

## Abstract

Recently several reports have demonstrated that innate immune response and inflammation have an important role in major neurodegenerative diseases. The activation of the NF-κB family of transcription factors is a key step in the regulation of pro inflammatory cytokine expression. Microglia and other cell types in the brain can be activated in response to endogenous danger molecules as well as aggregated proteins and brain injury. During the past couple of years several studies reported the role of cystatins in neuroinflammation and neurodegeneration. In the present review, I will summarize and analyze recent findings regarding the role of cystatins in inflammation and NF-κB activation. Type I cystatin stefin B (cystatin B) is an endogenous cysteine cathepsin inhibitor localized in the cytosol, mitochondria and nucleus. Mutations in the gene of stefin B are associated with the neurodegenerative disease known as Unverricht-Lundborg disease and microglial activation plays an important role in the pathogenesis of the disease. Stefin B deficient mice have increased caspase-11 expression and secreted higher amounts of pro-inflammatory cytokines. The increased caspase-11 gene expression, was a consequence of increased NF-κB activation.

## Introduction

The innate immune response represents not only the first line of defense against invading microorganisms, but also responds to endogenous stress and has a crucial role in the pathology of neurodegenerative diseases in the central nervous system (CNS). It is elicited through the detection of pathogen-associated molecular patterns (PAMPs) and danger-associated molecular patterns DAMPS (Akira et al., 2006; Medzhitov, 2007). In the CNS, pattern-recognition receptors (PRRs) are primarily expressed by microglia and astrocytes. DAMPs are endogenous molecules, released by dying or damaged cells after cellular stress and can be recognized by PRRs such as membrane-bound toll-like receptors (TLRs) or cytosolic nucleotide-binding domain and leucine-rich repeat-containing (NLR), the RIG-I-like receptor (RLR), the AIM2-like receptor (ALR; Medzhitov, 2007; Moresco et al., 2011; Franchi et al., 2012). Triggering of PRRs by PAMPs or DAMPs results in signaling pathways that promote gene transcription by nuclear factor-κB (NF-κB) as well as interferon regulatory factors (IRFs) and leads to production of interferons and pro-inflammatory cytokines (Akira et al., 2006; Kawai and Akira, 2009). TLR4 and the extracellular adaptor protein MD-2 form a lipopolysacharide (LPS) receptor complex. Upon LPS binding and receptor homodimerization, the signal transducing adapter proteins become associated with the receptor and trigger the signaling pathway resulting in the activation of the nuclear NF-κB (Visintin et al., 2003; Medzhitov, 2007). With the exception of TLR3, TLRs and the IL-1R family use the adaptor MyD88 for signal transduction, which recruits the kinases}IRAK1 and IRAK4 as well as TRAF6 through its death domain (Muzio et al., 1997; Wesche et al., 1997; Kobayashi et al., 2002). The result of these sequential steps is the activation of inhibitors of NF-κB kinase (IKK) complex and the transcription factor nuclear factor kappa B (NF-κB). Viral PAMPs, like nucleic acids are recognized by endosomal TLRs −3, −7, −8 and −9, as well as by RLRs. Triggering endosomal TLRs by viral PAMPs leads to the activation of NF-κB and IRFs, which cooperatively mediate the production of IFN-α/β (Apostolou and Thanos, 2008). In the CNS, pattern-recognition receptors are primarily expressed by microglia, macrophages and astrocytes.

A crucial component of the innate immune response is the inflammasome, a NLR-based multiprotein complex responsible for the activation of caspase-1 (Martinon and Tschopp, 2007; Latz et al., 2013; Lamkanfi and Dixit, 2014). Members of the NLR family (NLR proteins –NLRP) and the adaptor ASC form multiprotein complexes which are required for the activation of the pro-inflammatory caspase-1 and subsequent processing of pro-interleukin (IL)-1β and pro-IL-18 into the mature forms that are released from the cell (Martinon and Tschopp, 2007; Takeuchi and Akira, 2010). The inflammasome activation should be tightly regulated in order to protect the host and limit the excessive inflammation. Recently, several excellent reviews described mechanism of inflammasome activation (Lamkanfi and Dixit, 2014; Guo et al., 2015). Particullary the activation of NLRP3 inflamamsome is well characterized (Martinon and Tschopp, 2007; Jabaut et al., 2013; Latz et al., 2013). The nucleotide binding and oligomerization domain-like receptor family pyrin domain containing 3 (NLRP3) inflammasome, is composed of NLRP3, the adaptor molecule apoptosis-associated speck-like protein which contain a caspase recruitment domain (ASC) and the cysteine protease caspase-1. The first, priming step is provided by TLR signaling that up-regulates NLPR3 and pro-IL-1β gene expression. This process is tightly controlled by signals culminating in the activation of NF-κB (Bauernfeind et al., 2009).

Recently not only canonical NLRP3 inflammasome activation (LPS and ATP), but also non-canonical inflammasome activation was reported (Kayagaki et al., 2011; Rathinam et al., 2012; Broz and Monack, 2013). Canonical inflammasomes activation results in procaspase-1 cleavage and activation, whereas the activation of a noncanonical inflammasome results in activation of procaspase-11 (Lamkanfi and Dixit, 2014). The canonical and noncanonical inflammasomes regulate release of IL-1α and IL-1β (Kayagaki et al., 2011). Both proinflamatory caspases-1 and -11 could induce pyroptosis, however only caspase-1 processes proforms of cytokines IL-1β and IL-18 into active forms which are secreted (Kayagaki et al., 2011). Recently two independent reports demonstrated that gasdermin D, a substate for caspase-11, was essential for caspase-11-dependent pyroptosis and interleukin-1β maturation (Kayagaki et al., 2015; Shi et al., 2015). Caspase-11 responds to cytoplasmic LPS independent of Toll-like receptor 4 (Hagar et al., 2013; Kayagaki et al., 2013). The mouse caspase-11 has high similarities to caspase-1 and is orthologous to human caspases-4 and -5 (Wang et al., 1996; Kajiwara et al., 2014). Interestingly, caspase-11-deficient mice, but not caspase-1-deficient mice are partially protected from septic death (Wang et al., 1998; Kayagaki et al., 2011).

In neurons NLRP1 and Aim2 inflammasomes were described (de Rivero Vaccari et al., 2009; Adamczak et al., 2014) and in microglia microglia NLRP3 (Halle et al., 2008), NLRP2 inflammasome was reported in human astrocytes (Minkiewicz et al., 2013). Recent study by Adamczak et al. (2014) reported that activation of the Aim2 inflammasome in neurons by cerebrospinal fluid from traumatic brain injury patients resulted in neuronal pyroptosis. Recently, several reviews described the role of innate immune response and inflammasome activation in neurodegenerative diseases (Heneka et al., 2014, 2015; Freeman and Ting, 2015). The CNS is particularly sensitive to IL-1β and IL-18 signaling because several cell types in the CNS express receptors for these cytokines (Allan et al., 2005; Alboni et al., 2010). Recently, several reviews described the role of innate immune response and inflammasome activation in neurodegenerative diseases (Heneka et al., 2014, 2015; Freeman and Ting, 2015).

## Microglia and Astrocytes—Immune Cells in the Brain

In CNS, the blood-brain barrier (BBB) limits entry of peripherally derived innate and adaptive immune cells and their associated inflammatory molecule and plasma proteins (Banerjee and Bhat, 2007). Moreover, the CNS environment is anti-inflammatory, featuring high local concentrations of inflammation-suppressive cytokines such as TGF-β and IL-10 (Zocchia et al., 1997). Microglia are CNS-resident myeloid cells of embryonic hemeatopoietic origin and represent a major cellular component of the innate immune system in CNS (Aguzzi et al., 2013). Similar to tissue macrophages, microglia survey the brain for pathogens and support CNS homeostasis and plasticity; by guarding and remodeling synapses (Wake et al., 2009; Parkhurst et al., 2013). Other cell types in the brain, endothelial cells, astrocytes, and neurons, can also contribute to inflammatory responses in the brain. They also express innate immune receptors and can directly respond to DAMPs or PAMPs. In a resting state microglia cells have a ramified morphology, they have a large number of processes that enable the interaction of microglia with neurons and astrocytes. They search for dysfunctional synapses, which they are able to eliminate by phagocytosis (Wake et al., 2009; Tremblay et al., 2010). After immunological stimuli, like viral infection or brain injury microglia cells are activated (Davalos et al., 2005; Nimmerjahn et al., 2005) and acquire a compact phenotype. They upregulate several surface receptors, like receptors for neuro-transmitters, cytokines and chemokines, as well as PRRs (Rock et al., 2004; Block et al., 2007). Several TLRs are expressed on microglial surface, including TLR2, TLR4 and TLR6 (Udan et al., 2008; Stewart et al., 2010; Fellner et al., 2013). Activation of TLR4 together with CD14 is implicated in brain inflammation and microglial activation in response to sepsis (Chakravarty and Herkenham, 2005). The activation of microglia also leads to production of reactive oxygen species (ROS) through the induction or activation of NADPH (Shimohama et al., 2000) and NO (Vodovotz et al., 1996; Heneka et al., 2001).

Astrocytes provide neuronal support in healthy conditions and can undergo several phenotypic changes that could be protective or causative with regard to pathology (Sofroniew and Vinters, 2010). For the difference from microglia astrocytes descend from neuroepithelial stem cells. Upon triggering of TLR, astrocytes participate in innate immune reactions and secrete inflammatory mediators, like complement components, IL-1β and IL-6. It was reported that astrocyte exposure to LPS switches astrocyte of pro-inflammatory genes expression (Hamby et al., 2012; Zamanian et al., 2012). Interestingly, the ischemeia caused by experimental stroke *in vivo* shifts the astrocyte transcriptome towards neuroprotective mechanisms (Zamanian et al., 2012). Although astrocytes may undergo distinct phenotypic changes and secrete pro-inflammatory cytokines, recent study demonstrated that NLRP3 inflammasome was not functional in mouse astrocytes, but only in microglia cells (Gustin et al., 2015). However, it was reported that in human astrocytes NLRP2 inflammasome was activated by the ATP and the procersing of caspase 1 and IL-1β was confirmed (Minkiewicz et al., 2013). Activation of innate immune receptors, as well as inflammasome activationa and IL-1β release activate NF-κB signaling.

## NF-κB

Nuclear factor kappa B (NF-κB) was first described by David Baltimore, as an inducible transcription factor in lymphocytes (Sen and Baltimore, 1986). In the CNS, NF-κB signaling is essential in several of physiological as well as pathological processes associated with neurodegeneration (Kaltschmidt et al., 1997; Ghosh et al., 2007; Crampton and O’Keeffe, 2013). The NF-κB family comprises several transcription factors that contain Rel-homology domains (RHDs) that bind to specific DNA sequences known as κB sites present in promoter and enhancer regions of various genes (Hayden and Ghosh, 2012). In mammalian cells five NF-κB factors were described: RelA (p65), RelB, c-Rel, p105 (NF-κB1; a precursor of p50) and p100 (NF-κB2; a precursor of p52; May and Ghosh, 1997). Homo- and heterodimers dimers could be formed through N-terminal DNA-binding/dimerization domain, known as the Rel homology domain. NF-κB dimers can modulate gene expression by binding to a variety of DNA sequences called κB sites (Karin, 2009). RelB, c-Rel, and p65 contain C-terminal transcription activation domains that enable recruitment of co-activator and target gene expression. p50 and p52 could form heterodimers with p65, c-Rel, or RelB and activate transcription of target genes. In addition, when they form homodimers, they repress transcription on binding to DNA (Hayden and Ghosh, 2011). In resting cells, NF-κB complexes are inactive, located mainly in the cytoplasm in a complex with IκB proteins. The IκB inhibitors contain ankyrin repeats and seven different inhibitors were described: IκBα, IκBβ, Iκ*B*ε, Iκ*B*ζ, p100, p105, Bcl3, IκBns. IκBα, IκBβ, IκBγ and Iκ*B*ε can hide the nuclear localization signal of NF-κB in the cytoplasm by direct interactions and this way prevent nuclear translocation of NF-κB. On the other hand, Bcl-3, Iκ*B*ζ and IκBNS are present in the nucleus and interact with NF-κB to regulate transcription (Hayden and Ghosh, 2008; Karin, 2009). Upon activation of innate receptors and signaling the rapid phosphorylation of specific serine residues of IκB proteins by a multiprotein complex termed the IKK complex occurs. IKK complex consists of two catalytically active kinases, IKKα and IKKβ, and a regulatory protein NEMO (NF-κB essential modifier, also known as IKKγ). IKKα and IKKβ are structurally similar and have a kinase domain, a leucine zipper domain, helix–loop–helix structures and a NEMO-binding domain (NBD). The IκB proteins are degraded by proteasome and NF-κB dimers enter the nucleus. IκBβ plays a unique role in determining specific target gene expression; it preferentially binds cRel-containing NF-κB dimers, and these dimer combinations bind to specific DNA sequences and selective downstream genes are targeted (Sen and Smale, 2010). Several different stimuli could trigger NF-κB signaling in the CNS, including cytokines (tumor necrosis factor κ (TNFα) and IL-(1), chemokines, virus as well as injury or oxidative stress. In some cases stimuli are specific for a cell type.NF-κB is activated by stimuli specific of the CNS like neural cell adhesion molecule (N-CAM; Krushel et al., 1999), neurotrophins (NGF and S100ββ; Carter et al., 1996) or amyloid β (Aβ) peptide (Behl et al., 1994). NF-κB enables the transcription of the genes encoding many pro-inflammatory cytokines and chemokines. Since cells from *Nemo*-deficient mice do not exhibit NF-κB activation by LPS or IL-1 (Rudolph et al., 2000), activation of NF-κB responsive genes by the innate immune triggers depends on NEMO and progresses through the canonical NF-κB signaling pathway. The expression of inducible NO synthase (iNOS), that mediates NO production, is regulated by NF-κB and STAT transcription factors (Farlik et al., 2010). The targets of NF-κB-dependent pro-inflammatory cytokines, such as TNFα, tend to be receptors that in turn, activate NF-κB pathway. NF-κB is critical to the propagation and elaboration of cytokine responses. TNF-α plays an important role in both local and systemic inflammation, and it is a powerful inducer of NF-κB. The predominant pathway triggered by TLR signaling is the canonical NF-κB pathway, where IKKβ-dependent phosphorylation of IκBα or IκBβ results in their ubiquitination and degradation by the proteasome (Kawai and Akira, 2007). The nuclear NF-κB dimers bind to κB DNA sites and start a transcriptional program that includes numerous effectors of the innate immune system. In the alternative (or non-canonical) pathway, both p105 and p100 contain C-terminal ankyrin repeats that function as IκB-like proteins. The p100-RelB complex is activated by phosphorylation of the C-terminal region of p100 by an IKKα–IKKα homodimer (lacking IKKβ and NEMO), which leads to ubiquitination and further degradation of the p100 IkB-like C-terminal sequences to generate a p52-RelB heterodimer (Sun, 2011). This pathway is triggered by CD40, LT (lymphotoxin) β receptor and the BAFF (B-cell-activating factor belonging to the TNF family) receptor.

In either pathway, the unmasked NF-kB complex can then enter the nucleus to activate target gene expression. In the classical pathway, one of the target genes activated by NF-κB is that which encodes IkBα. Newly-synthesized IkBα can enter the nucleus, remove NF-κB from DNA, and export the complex back to the cytoplasm to restore the original latent state. Furthermore, IKKα-deficient mice show increased production of pro-inflammatory chemokines and cytokine and show increased inflammatory responses in local and systemic inflammation (Lawrence et al., 2005; Li et al., 2005). The IKKα-mediated repression of transcriptional responses influence the level of nuclear p65 and c-Rel (Lawrence et al., 2005). Recent reports have shown that IKKβ also influence anti-inflammatory response in macrophages. IKKβ not only inhibits macrophage function by interfering with the STAT pathway during infection (Fong et al., 2008), but also suppresses the secretion of IL-1β (Greten et al., 2007). In the CNS, NF-κB activation can be negatively regulated by a several cytokines like IL-4, IL-10, transforming growth factor β (TGFβ), and other molecules like glycogen synthase kinase-3 (GSK-3β) and glucocorticoids (Kaltschmidt et al., 2005). During neuro inflammation in activated glial cells IL-4 inhibits NF-κB via a peroxisome proliferator activated receptor (PPAR)-γ-mediated mechanism and allows survival of differentiating oligodendrocyte precursors (Paintlia et al., 2006). Other studies demonstrated that GSK-3β negatively regulated NF-κB activity in astrocytes (Sanchez et al., 2003). Moreover, it was reported that IL-4 and IL-10 that block IL-1β-induced NF-κB activation in astrocytes, also diminished IL-1β-induced Akt phosphorylation (Pousset et al., 2000).

## Cystatins

**Figure 1:**
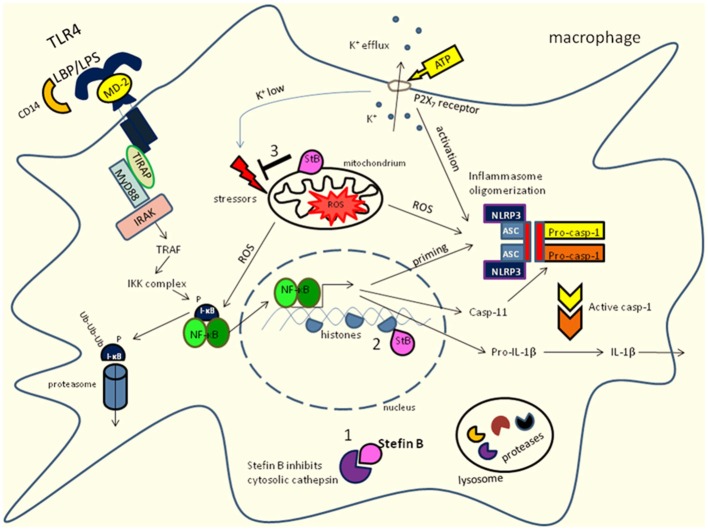
**Innate receptor signaling and stefin B influence NF-κB activation and caspase-11 expression in macrophages.** Stefin B could act an inhibitor of cysteine cathepsins in cytosol (1) and nucleus (2) in mitochondria (3) prevents the excessive ROS formation. Toll-like receptor (TLR) signaling is activated by TLR ligands. Signaling through TLR4 which is located in plasma membrane activate NF-κB via MyD88 dependent pathway. Moreover, caspase-11 expression is regulated by NF-κB signaling. In addition, LPS stimulation results in increased mitochondrial ROS formation. LPS stimulation could lead to non-canonical inflammasome activation of caspase-11 and caspase-1 that results in IL-1β and IL-18 processing and secretion.

Cystatin is a name derived from cysteine protease inhibitor. They are reversible and tight-binding inhibitors of the papain (C1) and legumain (C13) families of cysteine proteases and have significant similarities in amino acid sequence (Barrett, 1981; Barrett et al., 1986). The MEROPS database (http://merops.sanger.ac.uk/) lists members of the cystatin family (family I25, clan IH), distributed in protozoa, plants, fungi and animals as well as in viruses (Rawlings et al., 2014). Initially, they were characterized as inhibitors of endolysosomal cysteine cathepsins, however recently some alternative functions for cystatins were proposed. Endolysosomal proteases and their inhibitors play an important role in the initiation and regulation of immune response (Bird et al., 2009; Kopitar-Jerala, 2012). On the basis of sequence homology, the presence or absence of disulfide bonds and physiological localization, of three families of cystatins were described: family I (stefins), family II (cystatins) and family III (kininogens; Kopitar-Jerala, 2006; Turk et al., 2008). Type 1 cystatins—stefins are mostly intracellular cystatins present in the cytosol, mitochondria and the nuclei (Abrahamson et al., 1986; Ceru et al., 2010; Maher et al., 2014a). Type 2 cystatins are mainly extracellular, secreted proteins. They are synthesized with 20–26 residue long signal peptides and most of them are found in physiologically relevant concentrations in body fluids (Abrahamson et al., 1986; Kopitar-Jerala, 2006; Turk et al., 2008). Type II cysatins possess also a second reactive site for inhibition of the C13 family of cysteine proteases (legumain; Alvarez-Fernandez et al., 1999). Cystatin C is expressed in a variety of tissues and its expression and localization was associated with various neurodegenerative pathologies like Alzheimer’s disease (AD; Kaur and Levy, 2012). It associates with Aβ and inhibits Aβ oligomerization *in vitro* and *in vivo* (Mi et al., 2007). A point mutation in the cystatin C gene is responsible for the dominantly inherited icelandic type of amyloidosis, hereditary cystatin C amyloid angiopathy (HCCAA; Olafsson and Grubb, 2000). The increased expression of cystatin C was observed in response to different types of insults to the brain, such as ischemia and epilepsy (Palm et al., 1995; Lukasiuk et al., 2002). Another type II cystatin, cystatin F was found abundant in the cells of the immune system: myeloid cells (macrophages and dendritic cells) and the cells involved in target cell killing (NK cells and cytotoxic T cells (CTLs; Halfon et al., 1998; Ni et al., 1998; Obata-Onai et al., 2002). It is expressed as di-sulfide-linked dimer (Cappello et al., 2004) and translocated to endolysosomes where it regulates cathepsin activity. Cystain F transport to endolysosomes depends on its *N*-linked glycosylation and it was reported that the secreted dimeric cystatin F could be internalized and activated by the mannose-6-phosphate receptor system (Colbert et al., 2009). Cleavage of its N-terminal region by cathepsin V leads to the monomerization and influence the inhibitory properties (Maher et al., 2014b). After proteolytic removal of its N-terminal part, cystatin F becomes a potent inhibitor of cathepsin C with the potential to regulate pro-granzyme processing and cell cytotoxicity (Hamilton et al., 2008). In cytotoxic cells, cystatin F therefore appears as a key regulator of granzyme processing and consequently cell cytotoxicity. Cystatin D was reported to inhibit proliferation, migration and invasion of carcinoma cells (Alvarez-Díaz et al., 2009). In addition, it was shown that a proportion of cystatin D is targeted to cell nucleus at specific transcriptionally active chromatin sites (Ferrer-Mayorga et al., 2015). Type-3 cystatins are high molecular weight (60–120 kDa) proteins and have three repeated type 2-like cystatin domains (Salvesen et al., 1986). Cystatins in immune cells have been reported to participate in the release of NO, phagocytosis and expression of cytokines (Kopitar-Jerala, 2006; Magister and Kos, 2013; Maher et al., 2014a). Stefin B belongs to the type one cystatins and is located in the cytosol, mitochondria and nucleus (Ceru et al., 2010; Maher et al., 2014a). Mutations in the gene encoding stefin B are present in patients with a form of progressive myoclonus epilepsy of Unverricht-Lundborg type (EPM1; Pennacchio et al., 1996, 1998; Lalioti et al., 1997a, b). EPM1 is an autosomal recessively inherited neurodegenerative disease, characterized by the cerebellar granule neurons apoptosis, progressive ataxia and myoclonic epilepsy (Pennacchio et al., 1996; Joensuu et al., 2008). In lymphoid cells of EPM1 patients, increased cathepsin activity, due to reduced expression of stefin B was reported (Rinne et al., 2002), we determined increased overall cathepsin activity in untreated, as well as in classically activated stefin B-deficient BMDMs compared to wild type cells (Maher et al., 2014c). We showed that in the nucleus stefin B interacts with cathepsin L and nucleosomes, specifically with histones H2A.Z, H2B, and H3 (Ceru et al., 2010). Goulet et al. (2004) has shown that only shorter procathepsin L isoforms translocate to the nucleus and stimulate processing of the CUX1 transcription factor at the G_1_/S transition of the cell cycle. The delay in cell cycle progression in cells overexpressing stefin B was associated with the inhibition of cathepsin L in the nucleus, confirmed by decreased cleavage of the CUX1 transcription factor (Ceru et al., 2010). In addition, we have shown that stefin B overexpression in the nucleus of astrocytoma cells T89G delayed not only cell cycle progression, but also caspase activation (Sun et al., 2012). A recent work demonstrated that the early microglial activation precedes neuronal loss in the brain of the stefin B deficient mice (Tegelberg et al., 2012). In our recent work we showed that stefin B-deficient mice were significantly more sensitive to the lethal LPS-induced sepsis due to increased caspase-11 expression (Maher et al., 2014a). The increased caspase-11 gene expression and better caspase-1 and -11 processing determined in stefin B deficient bone marrow-derived macrophages (BMDMs) resulted in enhanced IL-1β and IL-18 processing and secretion (Figure 1). Upon LPS stimulation, stefin B was targeted into the mitochondria, and the lack of stefin B resulted in the increased destabilization of mitochondrial membrane potential and mitochondrial ROS generation. We have determined increased NF-κB signaling in stefin B deficient BMDMS upon LPS stimulation, in reverse experiment when stefin B was over-expressed in macrophage reporter cell line RAW-blue, a decreased NF-κB activation was determined upon LPS stimulation (Maher et al., 2014a). The expression of caspase-11 is regulated by NF-κB and STAT-1 transcription factors (Schauvliege et al., 2002). Since STAT-1 signaling is downregulated in stefin B deficient BMDMs upon LPS stimulation. We propose that stefin B could modulate the caspase-11 gene expression in an NF-κB-dependent manner. Okuneva et al. (2015) reported significantly higher stefin B mRNA expression in microglia than in neurons or astrocytes, which is in line with our observation that stefin B is highly upregulated in activated macrophages (Maher et al., 2014c). In stefin B-deficient macrophages we detected increased LPS-induced pro-inflammatory NO production, but decreased IL-10 expression (Maher et al., 2014c). The phosphorylation of ERK and JNK MAP-kinases were significantly decreased in stefin B-deficient macrophages, as well as STAT-3 phosphorylation. The results in macrophages could be compared to the signaling events in microglia. Activation of cocultures of microglial and astroglial cells with LPS strongly induces IL-10 mRNA expression, IL-10 production and release (Ledeboer et al., 2002).

Among type II cystatins, the most abundant cystatin is cystatin C. It was described first as a “post-γ-globulin” or “γ-trace” (Grubb and Löfberg, 1982). It is expressed in various tissues and mostly secreted from the cells (Abrahamson et al., 1986). In the CNS, cystatin C is secreted from the choroid plexus into the cerebrospinal fluid. Various studies showed that cystain C has a neuroprotective role in neurodegenerative diseases (Mi et al., 2007; Kaur and Levy, 2012). Frendéus et al. (2009) reported that mouse peritoneal macrophages lacking cystatin C expressed higher levels of IL-10 mRNA but lower TNF-α upon IFN-γ stimulation, compared to similarly primed wild type cells. The cystatin C-deficient macrophages also have reduced NF-κB p65 activation, compared to control cells. The precise mechanism by which cystatin C influence increased IL-10 expression is still elusive. Electrophoretic mobility shift analysis of nuclear extracts from macrophages and showed that IL-10 suppressed nuclear localization of NF-κB (Lentsch et al., 1997). Increased IL-10 expression could suppers NF-κB activation in cystatin C deficient cells.

## Conclusions

The precise mechanism by which type I cystatins, like stefin B and type II cystatins cystatin C influence is still not clear. The type one cystatins, stefins A and B are localized to the cytoplasm and nucleus, while type II cystatins are predominately secreted. In addition, upon LPS stimulation stefin B is targeted to mitochondria and prevents excessive mitochondrial ROS formation. The lack of stefin B resulted in increased mitochondrial ROS formation and an increased NF-κB activation, as well as the expression of NF-κB target genes. Increased expression of stefin B in macrophages resulted in diminished NF-κB signaling, while exogenous addition of cystatin C to macrophages enhanced NF-κB p65 activity and NF-κB target genes expression. Further experiments on cell cultures as well as on mouse models could help us to elucidate the precise mechanism by which cystatins influence NF-κB signaling. Better understanding of the role of cystatins in these signaling pathways could lead also to the development of new therapies and medications.

## Conflict of Interest Statement

The author declares that the research was conducted in the absence of any commercial or financial relationships that could be construed as a potential conflict of interest.
